# Using Personal‐Disclosure Mutual‐Sharing (PDMS) with first‐year undergraduate students transitioning to higher education

**DOI:** 10.1111/bjep.12502

**Published:** 2022-04-11

**Authors:** Andrew L. Evans, Matthew J. Slater, Martin J. Turner

**Affiliations:** ^1^ School of Health and Society The University of Salford Salford UK; ^2^ Centre for Sport, Health, and Exercise Research Staffordshire University Stoke‐on‐Trent UK; ^3^ Department of Psychology Manchester Metropolitan University Manchester UK

**Keywords:** coping, higher education, identification, personal‐disclosure mutual‐sharing, transition

## Abstract

**Background:**

Using Personal‐Disclosure Mutual‐Sharing (PDMS) with students transitioning to Higher Education (HE) has yet to be researched in education.

**Aims:**

In two studies, we aimed to explore the immediate effects of a Coping Oriented Personal‐Disclosure Mutual‐Sharing (COPDMS) intervention on first‐year undergraduate students’ relational and organizational identification, perceived social support availability, and self‐efficacy for learning and performance. In our second study, we also aimed to examine student‐perceptions of participating in a COPDMS intervention.

**Sample and Methods:**

At the beginning of induction week in both studies, first‐year undergraduate students on the same degree programme at a HE provider in England received an education session where COPDMS was introduced. Students participated in a COPDMS session a few days later. During COPDMS sessions, students mutually‐shared and disclosed personal information and/or stories relating to transitional experiences.

**Results:**

Across both studies, students’ relational identification with staff and perceived emotional, esteemed, and informational support availability from others on the degree programme significantly increased from pre‐ to post‐COPDMS phases. Findings relating to relational identification with other Year 1 students and perceived availability of tangible support were mixed. No significant changes occurred for organizational identification with the university and self‐efficacy for learning and performance. In Study 2, five higher‐order themes relating to students’ perceptions of COPDMS were found: (1) emotionality; (2) personal development; (3) storytelling; (4) enhanced group processes; and (5) task appropriateness and value.

**Conclusions:**

Study findings provide evidence that COPDMS is a useful psychological intervention to deliver to students transitioning to HE. Practical considerations, limitations, and future research suggestions are provided.

## BACKGROUND

Transitioning to Higher Education (HE) is challenging for students. For example, transitioning to HE requires students to establish new identities as HE learners (Briggs et al., 2012). Students can also struggle to relate to other students and staff (Briggs et al., 2012) and can experience difficulties envisaging university life (Smith & Hopkins, 2005). Transitions are regarded as a phase rather than a single event (Gale & Parker, 2012) which represent a period of preparing for, encountering, adjusting to, and stabilizing in a new environment (Nicholson & West, 1995). According to Nicholson’s (1990) Transition Cycle, students in the preparation phase develop expectations and desires to prepare for change. In the encountering phase, students attempt to make sense of their environment and cope with environmental demands. In the adjustment phase, students attempt to adjust in terms of personal change, roles, and relationships. The stabilization phase signals commitment and effectiveness and continues until preparation for the next transition occurs. Closely aligned to Nicholson’s (1990) Transition Cycle is Schlossberg’s (1981) Transition Theory, which postulates that a transition comprises three main components: (1) the experience leading up to the transition; (2) the evaluation of coping resources; and (3) taking charge of future transition to manage personal evolution. Within component two, Schlossberg (1981) proposed that an individual's potential to cope with a transition depends on the situation (e.g., previous transitional experiences), the self (e.g., psychological factors), support, and strategies/coping resources. Adaptive transitions occur when coping resources meet or exceed demand appraisals whilst maladaptive transitions occur when demand appraisals exceed coping resources. In this way, Schlossberg’s (1981) Transition Theory draws parallels with theories of stress appraisal (e.g., Cognitive Appraisal Theory; Lazarus, 1999) which emphasize that the relative balance or imbalance between demand and resource appraisals determines psychological, physiological, and behavioural responses.

To facilitate students’ transition to HE, universities have long‐implemented initiatives such as introductory courses (Hultberg et al., 2008) and induction activities involving group work to integrate students (Brooman & Darwent, 2014). Such initiatives tend to focus on information provision, academic tasks, or brief icebreakers. Drawing on Nicholson’s (1990) Transition Cycle, first‐year undergraduate students may benefit from sharing information about their transitional experiences with other students and staff members to facilitate their transition to HE whilst in the encountering phase. Personal‐Disclosure Mutual‐Sharing (PDMS) is a communication‐based intervention that would enable first‐year undergraduate students to personally‐disclose and mutually‐share information relating to their HE transitional experience. During PDMS interventions, individuals are tasked with preparing and sharing previously unknown personal information and/or stories to members of a group or team (Dunn & Holt, 2004). PDMS is derived from counselling settings where a client verbally presents a situation, issue, or aspiration to a practitioner and works collaboratively with that practitioner to gain resolution through interpersonal interaction (Holt & Dunn, 2006). The process of personal‐disclosure underpinned by mutual‐sharing has been posited to nurture empathy because individuals gain an enhanced understanding of one another's personal experiences (Dryden, 2006). PDMS has therefore been suggested to elicit change in individual (e.g., self‐confidence) and group constructs (e.g., closeness) through mechanisms that underpin person‐centred counselling approaches (e.g., Person‐Centred Therapy; see Rogers, 1951) where teamwork between a client and practitioner is therapeutic (Dryden, 2006). Researchers have also posited that the emotionality associated with listening to and disclosing information and stories elicits change in target variables (Barker et al., 2014). The emotionality of PDMS together with enhanced empathetic understanding has been suggested to strengthen socio‐emotional bonds between people, which may provide an additional theoretical mechanism underpinning PDMS outcomes. Finally, researchers have concluded that PDMS is a challenging experience (Evans et al., 2013). Undergoing a challenging intervention as a group or team may result in group or team members supporting one another through the intervention, which elicits change in outcome variables.

To date, PDMS research has been conducted in sports settings and has focused on the effects of four PDMS types. Each type of PDMS aims to elicit change in target variables identified in a needs analysis conducted by practitioners. The first type of PDMS is called Relationship Oriented PDMS (ROPDMS), which was introduced by Dunn and Holt (2004) as a team building intervention. ROPDMS involves individuals preparing and sharing personal information and/or stories outlining their character, motives, and desires in line with ROPDMS instructions (e.g., ‘tell the group why you play your sport and what you think you bring to the team?’). ROPDMS therefore focuses on integrating individuals and developing group dynamics. Qualitatively, research with teams comprising 27 male intercollegiate ice hockey athletes (Dunn & Holt, 2004) and 15 female high‐performance football athletes (Holt & Dunn, 2006) shows that ROPDMS enhances outcomes including understanding of the self and others and closeness. Quantitatively, ROPDMS has been found to significantly increase social identities and the value athletes place on friendships within their team in groups of 14 male football academy athletes (Evans et al., 2013) and 15 male academy cricketers (Barker et al., 2014). The second type of PDMS is called Mastery Oriented PDMS (MOPDMS) which involves individuals preparing and sharing personal information and/or stories in line with MOPDMS instructions around best performance (e.g., ‘Describe your best ever performance(s) in your sport’). MOPDMS therefore tasks athletes with sharing past performance accomplishments whilst they receive verbal persuasion information and vicariously experience success through the stories of others. Past performance accomplishments, verbal persuasion, and vicarious experiences are antecedents of efficacy (Bandura, 1997) and so, MOPDMS has been theorized to manipulate efficacy and achievement‐related variables (Barker et al., 2014). Indeed, Barker et al. found that collective‐efficacy and the value athletes placed on winning within their team significantly increased from pre‐MOPDMS to post‐MOPDMS intervention phases.

The third type of PDMS is called Rational Emotive PDMS (REPDMS) where individuals share personal experiences of adopting rational or irrational thinking following initial Rational Emotive Behaviour Therapy (REBT) education (Vertopoulos & Turner, 2017). Vertopoulos and Turner (2017) found that nine athletes who completed REPDMS following initial REBT education reported further reductions in irrational beliefs and increases in rational beliefs above and beyond those changes reported by 11 athletes who received REBT education only. Subsequent research by Turner and Davis (2019) in a sample of 23 triathletes found that REPDMS led to further increases in self‐determined motivation. The final type of PDMS is called Coping Oriented PDMS (COPDMS) which was developed by Evans et al. (2018) to support 18 youth academy football athletes with the prospect of transitioning within or out of their club. COPDMS involves individuals preparing and sharing information and/or personal stories relating to transitions whilst communicating demand and resource appraisals associated with transitions. Athlete insights revealed that COPDMS encouraged an approach focus and self‐confidence for upcoming transitions, altered cognitive appraisal about upcoming transitions, and increased understanding of self and others.

In sum, COPDMS has been used in a sporting context with athletes preparing for transition. Compared to other types of PDMS, COPDMS would therefore seem most appropriate to use with students transitioning to HE. Transitions are also a complex process comprising several demands, barriers, coping resources, outcomes, and consequences (Barclay, 2017). COPDMS tasks individuals with personally‐disclosing and mutually‐sharing information and/or personal stories relating to areas such as demands and coping resources. So, the task content of COPDMS aligns itself to the context of transition. Furthermore, coping is central to Nicholson’s (1990) Transition Cycle and Schlossberg’s (1981) Transition Theory. For example, the core of Schlossberg’s (1981) Transition Theory postulates that transition depends upon the situation, the self, support, and strategies/coping resources. Drawing on Transition Theory, it would therefore seem that transitioning to HE as a student requires significant coping potential. To this end, a COPDMS intervention for transition to HE would involve first‐year undergraduate students communicating demand and resource appraisals associated with transitional experiences. Based on theories of stress appraisal (e.g., Cognitive Appraisal Theory; Lazarus, 1999), increasing awareness of demand and resource appraisals pertinent to student transition to HE through COPDMS may develop a student's coping potential by helping them to meet or exceed transitional demands. Accordingly, theories of stress appraisal would suggest that promoting coping potential through COPDMS would facilitate the experience of adaptive outcomes.

### Our research context

Our idea of using COPDMS to assist first‐year undergraduate students transitioning to HE emerged from a meeting with staff members who taught on the same sports‐related degree programme at a HE institution in England. The meeting focused on contemporary issues relating to first‐year undergraduate students such as retention, progression, and the HE transition. At the time, central university data for the 2014/2015 cohort indicated that 8% of students withdrew from the degree programme during Year 1 following registration whilst 31% of students failed to progress from Year 1 to Year 2. Staff suggested that perhaps some students were not able to cope with their transition to HE which may have contributed to withdrawal and progression rates. During further discussions, staff highlighted that students’ relational identification with other Year 1 students and staff members, organizational identification with the university, perceived availability of social support, and self‐efficacy for learning and performance were particularly poor among previous Year 1 cohorts.

Relational identification represents the extent to which an individual defines themselves in terms of a given role‐relationship (Sluss & Ashforth, 2007). Research shows that relational identification influences students’ trajectories through education (March & Gaffney, 2010) and is therefore considered to be an important variable for student transition. Organizational identification refers to an individual's perception of belongingness and oneness with an organization (Ashforth & Mael, 1989) and positively predicts several outcomes within HE including student commitment, satisfaction, and achievement (Wilkins et al., 2015). Organizational identification has also been found to aid student transition (Lukács & Dávid, 2019). In organizational contexts, social support has been identified as a critical resource that enables individuals to cope with stressors (Halbesleben, 2006), which benefits academic, social, and emotional adjustment to HE (Pratt et al., 2000). Further research shows that organizational identification positively relates to perceived support (Sluss et al., 2008) and that those with a strong organizational identification receive more social support from others (Avanzi et al., 2018; Van Dick & Haslam, 2012). Finally, substantial evidence exists confirming the importance of students possessing self‐efficacy regarding their studies when transitioning to HE. For example, in a sample of 84 first‐year undergraduate students, Morton et al. (2014) found that self‐efficacy was a strong and positive predictor of student's adaptation to university. Theoretically, Morton et al. (2014) proposed that students who display high self‐efficacy regarding their studies may view themselves as able to meet the demands associated with their transition.

To conclude the team meeting, staff discussed strategies that could be implemented during induction week of the degree programme for future Year 1 cohorts to promote target variables. It was during this discussion that the first author suggested developing and delivering a COPDMS intervention to elicit improvements in target variables at the beginning of the degree programme.

Given our novelty of using COPDMS in education, we sought to examine the effects of COPDMS on a set of variables that were: (a) identified as psychological issues relating to transition in our needs analysis; and (b) highlighted in literature as being important for student transition. Specifically, across two studies, we aimed to explore the effects of COPDMS on relational and organizational identification, perceived availability of social support, and self‐efficacy for learning and performance. In Study 1, we report an initial investigation into the effects of COPDMS on target variables. In Study 2, we report a second investigation into the effects of COPDMS on target variables with a separate student cohort. Across studies, we hypothesized that first‐year undergraduate students’ relational identification with other Year 1 students and staff, organizational identification with the university, perceived availability of social support, and self‐efficacy for learning and performance would significantly increase from pre‐COPDMS to post‐COPDMS. In Study 2, we also sought to extend our initial investigations by exploring student perceptions of participating in a COPDMS intervention.

## STUDY 1

### Method

#### Participants and design

Fifty‐seven students (42 male) registered for the 2015/2016 sports‐related undergraduate degree programme (*M*age = 19.39 ± 2.68; range = 17–32 years). Thirty‐seven students provided quantitative data at pre‐ and post‐COPDMS phases, which were subject to statistical analyses.

Given our novelty of using PDMS in an educational context, we adopted an intervention design typically used by PDMS researchers (Dunn & Holt, 2004). Specifically, we administered a single‐bout of COPDMS through a one‐group pretest‐posttest design and focused on the immediate effects of COPDMS. Following recommendations by Windsor et al. (2011), the first author delivered the intervention given their relevant professional qualifications (British Association of Sport and Exercise Sciences Accredited psychology practitioner) and experience of delivering PDMS interventions. It was also deemed appropriate for the first author to deliver the intervention because they were the Year 1 manager.

At the beginning of induction week (Monday afternoon), the first author introduced COPDMS to students in a classroom before students completed a pre‐COPDMS questionnaire. The cohort was then randomly split into four COPDMS groups to: (a) ensure appropriate group sizes so all students could contribute to COPDMS sessions; and (b) avoid conducting overly long PDMS sessions which may lessen the impact of a PDMS session (see Evans et al., 2018; Windsor et al., 2011). Groups A and B completed their COPDMS session two days after the intervention was introduced (Wednesday morning). Groups C and D completed their COPDMS session three days after the intervention was introduced (Thursday morning) due to timetabling constraints and the first author's availability. Following each COPDMS session, students completed a post‐COPDMS questionnaire.

### The COPDMS questionnaire

Students rated the extent to which they agreed with all questionnaire items on a 7‐point scale ranging from 1 (*do not agree at all*) to 7 (*agree completely*).

#### Relational and organizational identification

We adapted two single‐item measures used in past research (Slater et al., 2018) to suit our study context to assess students’ relational identification with other Year 1 students (‘I identify strongly with other first year students’) and students’ relational identification with staff members (‘I identify strongly with my module tutors’). We also adapted a single‐item measure (Postmes et al., 2013) to suit our study context to assess students’ organizational identification with the university (‘I identify strongly with the university’).

#### Perceived social support availability

One item from each of the four subscales of the Perceived Available Support in Sport Questionnaire (PASS‐Q; Freeman et al., 2011) was used to measure students’ perceived availability of emotional, esteemed, informational, and tangible support from others on the degree programme. Specifically, students were asked to ‘rate the extent to which, if needed, someone on your degree programme would’ […] ‘care for you’ (emotional support), ‘boost your sense of competence’ (esteemed support), ‘give you advice about your university life’ (informational support), and ‘do things for you at university’ (tangible support). One item from each subscale was chosen to avoid overloading students with an extensive questionnaire and given time constraints associated with collecting data. The item selected from each subscale displayed the highest factor loading in validation research by Freeman et al. (2011) which were all acceptable.

#### Self‐efficacy for learning and performance

An 8‐item scale from the Motivation for Learning Strategies Questionnaire (the MLSQ; Pintrich et al., 1993) was used to measure students’ self‐efficacy for learning and performance (e.g., ‘I’m confident I can understand the basic concepts taught in this course’) and was found to be internally consistent at pre‐COPDMS (α = .90) and post‐COPDMS phases (α = .90).

#### Procedure

##### Stage 1: Ethical considerations

Institutional ethical approval was granted. Students read an information sheet before providing consent. In providing consent, students waived their right to anonymity and confidentiality because they would be participating in an intervention where information disclosed would be identifiable and accessible to others. Students consented that the only people not involved in the intervention that would receive information emanating from COPDMS sessions would be staff members unable to attend. In this way, staff members could still benefit from the intervention by receiving information that might enable them to better understand their students.

##### Stage 2: Initial introduction session

The first author explained that COPDMS would involve students personally‐disclosing and mutually‐sharing information relating to their transition to HE with other students and staff members. Furmark et al. (1999) found public speaking to be a common social phobia experienced by the age demographic of many of our students. Individuals can also feel apprehensive and express public speaking anxiety before PDMS sessions (Barker et al., 2014). The first author therefore attempted to alleviate any concerns about the intervention by referring to their experiences of delivering PDMS interventions. Students were also reassured that the process of sharing information through COPDMS was similar to sharing information during in‐class discussions commonly integrated within teaching and learning. A PDMS contract (Holt & Dunn, 2006) was subsequently presented to students on a projector screen to reinforce key ethical and procedural information. Like other PDMS contracts (Barker et al.), ours encouraged students to contribute to discussions, respect one another, listen, learn, and enjoy their PDMS experience. Our PDMS contract also reminded students about respecting and upholding anonymity and confidentiality. Since PDMS sessions involve social evaluation, students were reminded that delivering information was not a performance or test and that information disclosed would not warrant any preferential treatment or discrimination.

PDMS interventions typically involve presenting individuals with a set of instructions that guide the development of personal stories and/or information that are later delivered during a PDMS session (Dunn & Holt, 2004). PDMS practitioners will also commonly negotiate a speaking order for individuals to share their personal stories and/or information (Evans et al., 2013). We deviated from this traditional approach and instead informed students about themes they would explore during their COPDMS session because we wanted students to discuss their transitional experiences rather than deliver information in a pre‐determined order with no interaction. COPDMS themes were presented on a projector screen:

‘In preparation for your COPDMS session, consider the following: (1) previous transitions (changes) you have made (e.g., in education and sport), (2) demands or challenges associated with previous transitions, (3) resources (thoughts, feelings, and/or behaviours) that helped you with previous transitions, (4) your transition from college to university, (5) resources that will help you with the transition from college to university, and (6) what you have learnt from previous transitions that will help you with future transitions (e.g., in education and sport)’.

Each COPDMS theme was based on Schlossberg’s (1981) Transition Theory and theories of stress appraisal (e.g., Cognitive Appraisal Theory; Lazarus, 1999). Theme 1 prompted students to discuss situational factors relating to coping potential by mutually‐sharing information about past transitions. Theme 2 prompted students to articulate demand appraisals and further elucidate situational factors relating to coping potential by mutually‐sharing information about challenges associated with past transitions. Themes 3 and 5 prompted students to discuss resource appraisals relating to the self, support, and strategies/coping resources associated with adaptive previous and future transitions. Theme 4 prompted students to articulate their experience of transitioning from college to university, which explored component one of Schlossberg’s (1981) Transition Theory (the experience leading up to the transition). Finally, theme 6 prompted students to discuss future transitions which explored component three of Schlossberg’s (1981) Transition Theory (taking ownership of future transitions).

The session concluded with students having an opportunity to ask questions about their COPDMS session. Overall, the introductory session lasted around one hour.

##### Stage 3: COPDMS sessions

COPDMS sessions included the first author, Year 1 students, two Year 2 students, and two Graduate Teaching Assistants (GTAs). Year 2 students were present because they had recently been a Year 1 student and could therefore empathize with students. Empathetic understanding nurtured through PDMS has been suggested to underpin several individual and group‐level outcomes which we aimed to maximize through our intervention. Realizing that others have experienced or are experiencing similar events, thoughts, and feelings has also been found to help individuals understand more about themselves and others (Dunn & Holt, 2004), which we also aimed to maximize by including Year 2 students. GTAs were included because they would be teaching students during Year 1 modules. Discovering information from staff was anticipated to help students understand the nature of being a student on their degree programme. According to the Social Identity Approach (see Evans et al., 2016), understanding the value of group memberships influences the development of levels of identification by promoting self‐categorization, which we aimed to maximize through our intervention.

As is standard PDMS practice (see Holt & Dunn, 2006), the first author arranged chairs in a semi‐circle in a study room to encourage openness among students and staff members. On arrival, students were invited into the room and sat down. As is also standard PDMS practice (Evans et al., 2013), the first author reminded those present about the nature of the intervention and their PDMS contract. Students then spent a few moments reflecting on the COPDMS themes written on A4 paper. The first author began COPDMS sessions by inviting students to mutually‐share information relating to the first COPDMS theme. The first author facilitated disclosures and only moved onto the next theme once everyone in the room felt they had been provided with an opportunity to contribute. When no‐one initiated discussion, the first author moved around the semi‐circle sequentially and invited students from subgroups to disclose information. It was anticipated that someone within each subgroup would be willing to initiate discussion whilst this approach meant all students were continually provided with an opportunity to speak.

Following exploration of the final COPDMS theme, students were thanked for their time and commended for their honesty, openness, and willingness to engage. In‐keeping with previous PDMS sessions (Evans et al., 2013), a reflection around the key information disclosed during the COPDMS session was held. Specifically, the first author asked students and staff members to provide key points shared in relation to COPDMS themes. The first author also provided their own reflections. COPDMS sessions were recorded via a Dictaphone so the content of discussions could be later summarized by the first author. A written summary of information disclosed during COPDMS sessions across COPDMS groups was emailed to Year 1 students and staff at the beginning of the first teaching week. COPDMS sessions lasted approximately one hour.

#### Statistical analyses

Data from pre‐COPDMS and post‐COPDMS questionnaires were input into SPSS version 23. Scores with *z* values ±2*SD* were winsorized before potential changes in study variables from pre‐COPDMS to post‐COPDMS phases were explored.

### Results

#### Relational and organizational identification

No significant change in students’ relational identification with other Year 1 students was found from pre‐COPDMS (*md* = 6.00) to post‐COPDMS (*md* = 6.00; *Z* = 0.873, *p* > .05). Alternatively, students’ relational identification with staff members significantly increased from pre‐COPDMS (*md* = 5.00) to post‐COPDMS (*md* = 6.00; *Z* = 3.843, *p* < .01, *r* = .632). No significant change in students’ organizational identification with the university was found from pre‐COPDMS (*md* = 6.00) to post‐COPDMS (*md* = 6.00; *Z* = 0.040, *p* > .05).

#### Perceived social support availability

Significant increases from pre‐COPDMS to post‐COPDMS in students’ perceived availability of emotional (*md* = 5.00 vs. *md* = 6.00; *Z* = 2.241, *p* < .05, *r* = .374), esteemed (*md* = 5.00 vs. *md* = 6.00; *Z* = 3.076, *p* < .01, *r* = .513), and informational support (*md* = 5.00 vs. *md* = 6.00; *Z* = 3.193, *p* < .01, *r* = .532) were found. No significant change in students’ perceived availability of tangible support was found from pre‐COPDMS (*md* = 5.00) to post‐COPDMS (*md* = 6.00; *Z* = 0.825, *p* > .05).

#### Self‐efficacy for learning and performance

No significant change in self‐efficacy for learning and performance was found from pre‐COPDMS (*md* = 6.25) to post‐COPDMS (*md* = 6.25; *Z* = 1.404, *p* > .05).

Means and *SD*s for all variables at both phases are displayed in Table 1.

**TABLE 1:** Means and SDs for all variables at pre‐PDMS and post PDMS phases in Study 1

Variable	Pre‐PDMS, *M* ± *SD*	Post‐PDMS, *M* ± *SD*
Relational identification with other Year 1 students	5.54 ± 1.28	5.70 ± 1.08
Relational identification with staff	5.32 ± 1.00	6.14 ± 0.79**
Organizational identification with the university	5.81 ± 1.01	5.78 ± 0.96
Perceived emotional support availability	4.97 ± 1.11	5.42 ± 1.30*
Perceived esteemed support availability	4.94 ± 1.26	5.69 ± 1.09**
Perceived informational support availability	5.44 ± 1.11	6.08 ± 0.97**
Perceived tangible support availability	5.03 ± 1.25	5.19 ± 1.33
Self‐efficacy for learning and performance	6.24 ± 0.56	6.34 ± 0.48

*p < .05; **p < .01.

### Discussion

Students’ relational identification with staff members and perceived availability of emotional, esteemed, and informational support significantly increased from pre‐COPDMS to post‐COPDMS phases. However, no significant changes in relational identification with other Year 1 students, organizational identification with the university, perceived availability of tangible support, and self‐efficacy for learning and performance were found. Overall, Study 1 findings provide partial support for our hypothesis and provide preliminary evidence of the effects of COPDMS on target variables. In Study 2, we sought to further explore the effects of COPDMS on target variables with a separate student cohort. We also sought to extend our initial investigations by incorporating a qualitative element to our Study 2 methodology which enabled us to explore student‐perceptions of participating in a COPDMS intervention.

## STUDY 2

### Method

#### Participants and design

Fifty‐one students (47 male) registered for the 2019/2020 sport undergraduate degree programme. Twenty‐nine students (24 male; *M*age = 20.55 ± 7.51; range = 18–55 years) provided quantitative data at pre‐ and post‐COPDMS phases, which were subject to statistical analyses.

Like Study 1, the first author administered a single‐bout of COPDMS through a one‐group pretest–posttest design. Quantitative data were collected to explore the immediate effects of COPDMS whilst qualitative data were collected to explore student‐perceptions of participating in our COPDMS intervention. Like Study 1, the first author delivered the intervention given their relevant professional qualifications and experience of delivering PDMS interventions. The first author also remained in their role as Year 1 manager which served as further justification for them delivering the intervention.

At the beginning of induction week (Tuesday morning), the first author introduced COPDMS to students in a classroom before students completed a pre‐COPDMS questionnaire. Students where then randomly split into two COPDMS groups to ensure appropriate group sizes (see Evans et al., 2018). Groups A and B completed their COPDMS session two days later (Thursday afternoon). Following each COPDMS session, students completed a post‐COPDMS questionnaire. Students were also invited to participate in a focus group held five week's following each COPDMS session.

#### The COPDMS questionnaire

The COPDMS questionnaire contained the same measures used in Study 1. The internal consistency of the self‐efficacy for learning and performance measure was acceptable at pre‐COPDMS (α = .86) and post‐COPDMS (α = .90) phases.

#### Focus groups

Ten students agreed to attend a focus group. However, two students failed to show up. Focus group 1 therefore comprised three students and focus group 2 comprised five students. Each focus group included participants from PDMS group A and B so that student‐perceptions from both PDMS groups were captured. The first author conducted focus groups in a meeting room. To facilitate discussions, a semi‐structured focus group schedule was developed which invited participants to comment on: (1) the introduction to PDMS (e.g., ‘talk to me about what was going through your mind/what you were feeling when you were told you were going to complete a PDMS session?’), (2) preparing for PDMS (e.g., ‘how did you prepare for your PDMS session?’) and (3) the PDMS session (e.g., ‘talk to me about what it was like participating in your PDMS session?’; ‘tell me what you were thinking about/feeling during your PDMS session?’; and ‘talk to me about what you deem to be the benefits/drawbacks/limitations/challenges (if any) of participating in your PDMS session?’). Prompts were also used (e.g., ‘what more can you tell me about…?’) to further enhance the richness and depth of our data.

#### Procedure

##### Stage 1: Ethical considerations

Institutional ethical approval was granted. Students read an information sheet before providing consent. Information sheets and the informed consent form were structured like those used in Study 1, with the addition of information and consent around focus group participation.

##### Stage 2: Initial introduction session

The first author introduced the PDMS intervention in the same way as Study 1. So, the first author explained what COPDMS would involve, provided reassurance about sharing information, and presented the PDMS contract used in Study 1. Students were then presented with the COPDMS themes used in Study 1. The introductory session concluded with students being able to ask any questions. Overall, the introductory session lasted around one hour.

##### Stage 3: COPDMS sessions

COPDMS sessions included the first author, Year 1 students, and one Year 2 student. Unlike Study 1, no GTAs taught students during Year 1 modules which meant no GTAs were included within COPDMS sessions. In‐line with standard PDMS practice (see Holt & Dunn, 2006) outlined in Study 1, students and the first author sat on chairs arranged in a semi‐circle and were reminded about the nature of the intervention and PDMS contract. Students then reflected on the COPDMS themes. The first author began COPDMS sessions by inviting students to mutually‐share information relating to the first COPDMS theme. The first author facilitated disclosures as described in Study 1. Following exploration of the final COPDMS theme, students were thanked for their time and commended for their honesty, openness, and willingness to engage. PDMS sessions concluded with a reflective component where key information disclosed during the COPDMS session was reflected upon. COPDMS sessions were recorded via a Dictaphone and a written summary of information disclosed during COPDMS sessions was emailed to Year 1 students and staff at the start of the first teaching week. COPDMS sessions lasted around one hour.

#### Statistical analyses

Data from pre‐COPDMS and post‐COPDMS questionnaires were input into SPSS version 25. Scores with *z* values ±2SD were winsorized before potential changes in study variables from pre‐COPDMS to post‐COPDMS phases were explored.

#### Qualitative analyses

We conducted a six‐phase thematic analysis procedure outlined by Terry et al. (2017) in an iterative and recursive manner. In phase 1, the first author familiarized themselves with the data by transcribing data verbatim, reading and re‐reading transcripts, and noting initial observations. In phase 2, the first author generated initial codes by creating meaningful labels to segments of the dataset. The second author reviewed initial codes which led to the first author revisiting and refining codes on several occasions to clarify or modify earlier coding to generate coding consistency. In phase 3, the first author constructed themes by examining codes and combining, clustering, or collapsing codes into more meaningful patterns. The second author received iterations of constructed themes within tables which enhanced our ability to identify and understand potential themes in relation to each other and the overall dataset. In phase 4, the first author reviewed potential themes. In phase 5, the first author defined and named themes before writing‐up the analyses in phase 6.

### Results

#### Relational and organizational identification

Students’ relational identification with other Year 1 students significantly increased from pre‐COPDMS (*md* = 4.00) to post‐COPDMS (*md* = 6.00; *Z* = 3.830, *p* < .01, *r* = .711). Students’ relational identification with staff also significantly increased from pre‐COPDMS (*md* = 5.00) to post‐COPDMS (*md* = 6.00; *Z* = 3.260, *p* < .01, *r* = .605). No significant change was found in student's organizational identification with the university from pre‐COPDMS (*md* = 5.00) to post‐COPDMS (*md* = 5.00; *Z* = 1.684, *p* > .05).

#### Perceived social support availability

Significant increases from pre‐COPDMS to post‐COPDMS in students’ perceived availability of emotional (*md* = 4.00 vs. *md* = 5.00; *Z* = 3.684, *p* < .01, *r* = .684), esteemed (*md* = 4.00 vs. *md* = 6.00; *Z* = 4.039, *p* < .01, *r* = .750), informational (*md* = 5.00 vs. *md* = 6.00; *Z* = 3.213, *p* < .01, *r* = .600) and tangible support (*md* = 4.00 vs. *md* = 5.00; *Z* = 2.748, *p* < .01, *r* = .510) were found.

#### Self‐efficacy for learning and performance

No significant change in students’ self‐efficacy for learning and performance was found from pre‐COPDMS (*M* = 5.74 ± 0.63) to post‐COPDMS, *M* = 5.90 ± 0.65; *t*(28) = 1.816, *p* > .05.

Means and SDs for all variables at both phases are presented in Table 2.

**TABLE 2:** Means and *SD*s for all variables at pre‐PDMS and post PDMS phases in Study 2

Variable	Pre‐PDMS, *M* ± *SD*	Post‐PDMS, *M* ± *SD*
Relational identification with other Year 1 students	4.31 ± 1.37	5.72 ± 1.00*
Relational identification with staff	4.79 ± 1.35	5.55 ± 1.09*
Organizational identification with the university	5.07 ± 1.33	5.38 ± 1.08
Perceived emotional support availability	4.24 ± 1.12	5.10 ± 1.18*
Perceived esteemed support availability	4.52 ± 1.06	5.48 ± 0.95*
Perceived informational support availability	4.90 ± 1.50	5.76 ± 0.99*
Perceived tangible support availability	4.48 ± 1.41	5.07 ± 1.00*
Self‐efficacy for learning and performance	5.75 ± 0.63	5.90 ± 0.64

*p < .01.

#### Qualitative results

Qualitative data were abstracted into 19 lower order themes, and then, collapsed into five higher order themes (see Table 3): (1) emotionality; (2) personal development; (3) storytelling; (4) enhanced group processes; and (5) task appropriateness and value.

**TABLE 3:** Higher order and lower order themes from PDMS focus groups

Higher order themes	Lower order themes
1. Emotionality	Ready to go Interested Relaxed Uncertainty Feelings in relation to others Pride Relief
2. Personal development	Confidence Self‐reflection/understanding
3. Storytelling	Contributing Listening The environment
4. Enhanced group outcomes	Closeness Getting to know people Social support Communication
5. Task appropriateness and value	Applicability of transition focus PDMS groups Overall experience

##### Emotionality

The first higher order theme details the emotionality of the PDMS experience including seven lower order themes: ready to go, interested, relaxed, uncertainty, feelings in relation to others, pride, and relief. As lower order themes indicate, students expressed mixed emotions about their PDMS experience. The ‘ready to go’ lower order theme encompassed positive anticipatory feelings about PDMS (e.g., ‘I thought I’m here now and I’m in the chair this is it like we are only having a conversation. I was just looking forward to it [PDMS] more than anything’). Following the PDMS session, the ‘ready to go’ theme continued in the sense that some students expressed excitement and readiness to commence their studies. We also interpreted that some students were interested in doing PDMS (e.g., ‘I was genuinely intrigued about listening to people's stories’) which continued during the PDMS session (e.g., ‘It was fascinating listening to different people's stories and where different people came from’). Before and during the PDMS session, some students indicated they were ‘relaxed’ and ‘felt at ease’. However, not all students experienced positive emotions. For example, one student expressed uncertainty:

I was a bit on edge to be fair. For one, I’ve not spoken about that [transition] at all really and for two, I didn’t really know anyone else. So, I didn’t really know what to expect. I’ve never been in that situation before you know like in a big group speaking about things that I’ve never brought forward before.

Students’ uncertainties also centred around ‘knowing what to say’ and ‘when to speak’. Following the PDMS session, students experienced varied feelings in relation to their peers including feeling respect, humbled, and surprised. One student explained:

I came out of it [PDMS] with respect for people because some people spoke about their backgrounds. I know a couple of the lads come from disability backgrounds. You’ve got respect for people you know like where they’ve come from.

Meanwhile, another student said:

I haven’t really done anything quite like that before. I mean we’ve done it through school like the first class of the year you have to introduce each other. But it’s like say a fun fact. Whereas this [PDMS] was actually asking more detailed questions and you did come out of it [PDMS] with genuine empathy for other people.

We also found key patterns in the data reflected feelings of ‘pride’ as well as ‘relief because PDMS was over’.

##### Personal development

The second higher order theme details personal development in relation to PDMS including two lower order themes: confidence and self‐reflection/understanding. First, we interpreted from the data that students’ self‐confidence in speaking grew during their PDMS session (e.g., ‘My confidence was still growing even more so that I thought it would have done and I was inputting more’). Some students also recognized that others’ confidence grew (e.g., ‘A really good example was one of the girls from [city]. It's a completely different city, set‐up, background and environment and she's on her own. Towards the end you could see her confidence growing’). Following the PDMS session, some students experienced increased self‐confidence (e.g., ‘I was more confident in myself afterwards because two years ago I’d have never done anything like that’). The PDMS session also encouraged self‐reflection/understanding. For example, one student said:

You kinda review what you’ve been through and go wow I’ve actually been through these transitions. When you were in the session and you talk about them [transitions] you think aww I’ve actually been through a lot and I’ve got through it.

##### Storytelling

The third higher order theme details the process of storytelling within the PDMS session comprising three lower order themes: contributing, listening, and the environment. The contributing lower order theme encompassed views and experiences of stories told within the PDMS session. In particular, students highlighted that the first contribution set the tone for the session (e.g., ‘The first person that spoke was quite open and honest and I think that set the tone for the rest of the session’). Inspirational stories also significantly impacted people as exemplified by one student: ‘It nearly made me cry because you jump straight to ooh these are old people and university is for younger people. But him saying you can talk to me was just nice’. Although there were differences in the nature and extent of contributions, students felt everyone could contribute. Here, students felt that people should be ‘allowed to talk as long as they like’. Yet, students did highlight the negative repercussions of speaking too much. For example, one student said: ‘If one person dominates and talks for too long and too much then other people might not get the opportunity to say something or delve into what they want to say as much’. Whilst students acknowledged the importance of contributing (e.g., ‘If nobody contributes then it [PDMS] would just not work’), some students explained that it was useful to just listen to others tell their stories (e.g., ‘Even if you don't contribute you're still learning about everyone’). Key features of the environment that aided the storytelling process included feeling comfortable with the environment and the PDMS practitioner. Students also explained how ‘a level of maturity’ was needed within the room.

##### Enhanced group processes

The fourth higher order theme details enhanced group processes comprising four lower order themes: closeness, getting to know people, social support, and communication. First, we interpreted that students felt enhanced closeness following their PDMS session. Students explained that PDMS elicited ‘some kind of cohesion straight away’, made students ‘feel more connected’ and ‘was good for making friends’. Second, we interpreted that PDMS enabled students to get to know one another (e.g., ‘I’d say definitely benefits are you feel like you're getting to know people. You feel you know the people you're with a bit more because you know their experiences and you know the transition into where they are now’). By getting to know one another, students enhanced their understanding of one another (e.g., ‘I think it was helpful because you are learning about different people that you're gonna be spending quite a bit of time with on the course’). Third, we interpreted that PDMS promoted social support. One student explained:

To help yourself through university was to find a support system and that’s what we’ve done. We’ve found that now we’re all going through the same thing we can all help each other and we’ve all got mates. We’ve got a support system as well as the one we might have outside university. We’ve got another one inside university.

Finally, we interpreted that PDMS triggered further communication between students (e.g., ‘We had an hour and a half gap in‐between our sessions so we almost carried it [conversations] on and went into one of the cafés and carried on talking afterwards’). Some students also felt more comfortable with speaking to people in general following PDMS.

##### Task appropriateness and value

The fifth higher order theme details task appropriateness and value including three lower order themes: applicability of transition focus, PDMS groups, and overall experience. The lower order theme of applicability of transition focus encapsulated positive perceptions of the task content. Students deemed focusing on transition to be appropriate and relevant. To illustrate, one student said:

Oh it’s [transition] very relevant because we’re all going through a transition. Like my transition was coming from out of education to come back whereas people where making a transition who were stepping up. There were people who were obviously moving to here as well. So, transition was a very relevant theme because it would have been something we would have all been going through in various levels and degrees at that time. Well, still are really.

Some students also alluded to the novelty of sharing information about transition (e.g., ‘It's just not really the information that people usually share with each other in typical icebreakers’) and how they had ownership over what they shared. Students did have mixed views regarding PDMS groups. For example, some students talked about how PDMS groups limited understanding of the entire student cohort. For some students, increasing PDMS group sizes would have limited closeness. For other students, reducing PDMS group sizes would have increased how personal PDMS sessions felt. The overall experience encompassed favourable perceptions of the PDMS experience. Students explained that their PDMS experience was generally positive, worthwhile, and rewarding. One student advocated embracing the challenge of PDMS despite feeling anxious: ‘You'll feel the benefits of it [PDMS] afterwards. It was worth putting myself through the worry and the anxiety and everything to come out with what I came out with’. Another student commented that their PDMS session was the most memorable component of their induction week:

When I went home and my Mum was like oh how’s your first week at university been? That was the first time I kind of spoke about the fact that there were more people like me and if I think back to induction week if I’m honest that’s probably the most memorable thing about it. Like the other sessions we did were more like introductory sessions. But that’s [PDMS] the one I took the most from and that was the thing that epitomized the whole week.

### Discussion

Quantitative data revealed that students’ relational identification with other Year 1 students and staff and perceived availability of emotional, esteemed, informational, and tangible support significantly increased from pre‐COPDMS to post‐COPDMS phases. However, no significant changes in organizational identification with the university and self‐efficacy for learning and performance were found. Like Study 1, Study 2 findings provide partial support for our hypothesis and provide further evidence of the effects of COPDMS on target variables with another student cohort. Qualitatively, five higher order themes were identified within focus group data. Within the first theme (emotionality), students expressed feeling varied emotions (positive and negative) across the PDMS intervention. Within the second theme (personal development), students indicated experiencing enhanced confidence and increased understanding of themselves from undergoing their PDMS session. Within the third theme (storytelling), students explained the importance of contributing, listening and the environment. Within the fourth theme (enhanced group processes), students talked about how COPDMS elicited a positive change in a range of group‐level constructs. Within the final theme (task appropriateness and value), students highlighted the importance of focusing on transitions within a PDMS context and gave conflicting views around PDMS group sizes. That said, students’ views of their overall experience were favourable.

## GENERAL DISCUSSION

Across two studies, we explored the effects of COPDMS with first‐year undergraduate students on relational and organizational identification, perceived availability of social support, and self‐efficacy for learning and performance. In our second study, we also explored student‐perceptions of participating in a COPDMS intervention. Our research is the first to document the effects of PDMS and student‐perceptions of doing PDMS in an educational context. Our target variables have also not been studied in extant PDMS research.

In both studies, students’ relational identification with staff and perceived availability of emotional, esteemed, and informational support significantly increased from following COPDMS. Students’ relational identification with other Year 1 students and perceived tangible support significantly increased in Study 2 (but not Study 1). Qualitative data in Study 2 also confirmed that students felt increased social support following their COPDMS session. Taken together, these findings provide preliminary evidence that COPDMS benefits relational identification and perceived social support. Improvements in perceived social support may have been caused by increased relational identification. Research confirms that other types of identification (social) positively impacts social support (Haslam et al., 2016) but the causal associations between relational identification and social support remain unexplored and warrant further investigation. PDMS researchers have also theorized that mutual‐sharing strengthens socio‐emotional bonds between individuals (Hardy & Crace, 1997) because collaborative personal‐disclosure underpinned by mutual‐sharing nurtures empathetic understanding (Dryden, 2006). Indeed, qualitative data in Study 2 highlighted that students experienced enhanced closeness, understanding of one another, and empathy by undergoing COPDMS. Perhaps COPDMS triggered increases in relational identification and perceived social support through enhanced empathetic understanding and closeness, which could be explored as a potential theoretical mechanism in future research.

The Stress‐Buffering Model of social support (Cohen & Wills, 1985) could explain how COPDMS elicited individual (e.g., enhanced confidence) and group‐level outcomes (e.g., enhanced communication) within our research. The Stress‐Buffering Model theorizes that social support protects people from the potentially deleterious effects of stress, particularly when dimensions of social support are matched to the needs elicited by a stressful event (Cohen & Wills, 1985). COPDMS tasked students with completing a challenging task that required them to mutually‐share information that would be evaluated by others. Increased emotional and esteemed support may have helped students deal with such uncontrollable stressors associated with doing PDMS. Such social support fostered through COPDMS may have therefore helped students work through a stressful situation individually and collectively which perhaps gave rise to individual and group‐level outcomes. In contrast, increased informational and tangible support (in Study 2 only) may have helped students plan and prepare for the controllable aspects of their transition to HE which may have also elicited individual and group‐level outcomes. Exploring whether optimally matched social support drives changes in psychological outcomes in a PDMS context would be a useful future research endeavour.

Qualitative data in Study 2 indicated that students experienced a range of emotion throughout the COPDMS intervention which corroborates PDMS research in sport (Windsor et al., 2011) and highlights that PDMS is an emotional experience. Whilst students felt positive emotion, students also experienced negative emotion. For instance, some students felt uncertain about PDMS, which is comparable to past research (Dunn & Holt, 2004; Evans et al., 2018). Despite such uncertainty, quantitative and qualitative data suggests that COPDMS enhanced several outcomes. Researchers (Holt & Dunn, 2006) have advocated that the emotionality of PDMS should be embraced because an emotionally‐engaging experience may augment socio‐emotional bonds that lead to several individual‐level and group‐level outcomes. Indeed, qualitative data highlighted that embracing the challenge of PDMS as a student was worthwhile to do despite feelings of anxiousness. Perhaps the emotionality of PDMS also contributed to students experiencing enhanced individual and group‐level outcomes and is another potential theoretical mechanism that warrants further research attention.

Against our expectations, we found no change in students’ organizational identification with the university across our research. These findings may be attributable to information shared during COPDMS sessions focusing on factors relating to students, staff, and studying a degree rather than factors relating to the organization. Also against our expectations, we found no change in self‐efficacy for learning and performance across our research. Drawing on Bandura’s (1997) Self‐Efficacy Theory, COPDMS themes meant that students mutually‐shared information relating to successful transitions and accomplishments in education. Students also vicariously experienced success by listening to information, stories, and experiences about the educational accomplishments of others. Perhaps self‐efficacy for learning and performance did not change because COPDMS was not purely Mastery Oriented in nature. Additionally, students had not received any academic performance‐related information at the time of the intervention. Furthermore, COPDMS themes meant students mutually‐shared information, stories, and experiences relating to less successful transitions, which may have negated potential significant changes in self‐efficacy.

The storytelling theme identified within qualitative data in Study 2 has several implications for PDMS practitioners. First, students spoke about how initial contributions within COPDMS sessions set the tone for the session. This finding is consistent with PDMS research in sport (Holt & Dunn, 2006) illustrating that the first disclosure can set a president for those that follow. PDMS practitioners should therefore carefully consider which students initiate PDMS sessions. Second, students explained how inspirational stories had a meaningful impact. Encouraging students to provide emotional depth to what they disclose would seem to aid the emotionality of PDMS which could contribute towards the experience of important individual and group‐level outcomes. Third, students spoke about the significance of letting people speak but not having individuals dominate the discussion. Within traditional PDMS set‐ups where individuals take it in turns to deliver information and stories relating to PDMS themes, PDMS practitioners commonly provide guidance ahead of PDMS sessions around speech length and time (e.g., 2–3 min; Evans et al., 2013). However, our PDMS sessions involved students exploring themes sequentially. Being able to contribute at any point during the PDMS session was highlighted within data as being important which supports the suitability of facilitating PDMS sessions in this manner with student populations. Fourth, students explained that just listening to others was beneficial even if they were not contributing which is in line with past research (Barker et al., 2014). PDMS practitioners should therefore acknowledge that some students may not want to contribute but by being encouraged to attend and listen they can still have a beneficial PDMS experience. Finally, students spoke about how doing COPDMS in a mature and comfortable environment and being comfortable with their practitioner aided the storytelling process. Professional and mature environments have been suggested to facilitate disclosures previously (Evans et al.). PDMS practitioners should therefore promote professionalism and maturity and work with students to help them feel comfortable with doing PDMS.

The task appropriateness and value theme identified within qualitative data in Study 2 also has several implications for PDMS practitioners. Notably, students spoke to the importance of focusing on transition within a PDMS intervention and how PDMS encouraged students to share information not typically divulged in traditional icebreakers. PDMS was also cited in data as being a memorable experience. Whether participating in PDMS compared to other icebreaker activities (or nothing at all) is more memorable and beneficial could be investigated in future research. Moreover, students expressed conflicting views regarding PDMS group sizes. In sports settings, teams can comprise approximately five to 15 athletes which researchers suggest is an appropriate PDMS group size (Windsor et al., 2011). In educational settings though, student cohorts can be much larger which presents practitioners with a dilemma regarding group size. Research has found that subsequent bouts of PDMS can be less emotionally intense (Barker et al., 2014). Instead, practitioners could record PDMS sessions and share recordings with student cohorts so that everyone has access to information, stories, and experiences shared by all students. This may result in students understanding and being able to empathise and support their entire peer group rather than select individuals which may further elevate individual and group‐level outcomes.

For those seeking to do PDMS in education, we recommend that PDMS interventions are delivered by individuals who possess or are working towards (under supervision) accredited qualifications in a relevant psychological field. Being competent and qualified is important because PDMS sessions can involve sensitive information being disclosed by individuals, which requires practitioners to possess excellent counselling skills (Evans & Barker, 2020). Furthermore, a practitioner would need to sympathetically deal with challenges individuals may experience when engaging with PDMS such as uncertainty (Windsor et al., 2011). Examples of individuals in education who could administer PDMS interventions include qualified professionals, trained counsellors, welfare officers, psychologists, and postgraduate students under supervision.

### Limitations and future research

In Study 1, students received a COPDMS intervention. We then delivered the same COPDMS intervention to an alternate student cohort in Study 2 and found that the effects of COPDMS noted in Study 1 were largely replicated. Nevertheless, we fully appreciate that the lack of a control group in both studies threatens validity meaning our preliminary research findings should be interpreted with some caution. Having some students in a control group in an applied research study where the intervention being delivered was theorized as being useful for student transition into HE was deemed unethical and may have prevented students experiencing important individual and group‐level outcomes. It is also standard practice at universities to deliver activities or interventions to all students transitioning to HE. If the context provides the opportunity to do so, future researchers could improve the rigor of our design by using a multiple‐baseline single‐case design with multiple groups (see Letford & Gast, 2018) where the delivery of COPDMS is staggered. Staggering the delivery of COPDMS across groups would create control groups and overcome the ethical dilemma of having to withhold an intervention for control group participants (Barker et al., 2014). In our context, staggering the delivery of COPDMS was not possible because we had very limited time (three days) to conduct all induction activities. Such methodological dilemmas reflect the challenges of delivering PDMS interventions in this context and the tension between research and applied practice.

Students in both studies were at the beginning of their undergraduate degree programme and may have been socially desirable in their responses to be viewed favourably by others. Thus, future researchers could include the Marlowe‐Crowne Social Desirability Scale (Crowne & Marlowe, 1960) within questionnaires. Furthermore, it is plausible that students may have autonomously organized social activities or explored university services in‐between their introduction to COPDMS session and COPDMS session. To rule out such rival hypotheses and control for potential maturation effects, future researchers could send students introductory PDMS information prior to induction before implementing PDMS sessions at the onset of induction week. The challenge here is ensuring that students access and understand introductory information prior to their PDMS session.

Participants in our research were all studying the same degree programme at the same HE institution in one region of England. To maximize external validity, future researchers could investigate the effects of COPDMS and explore student‐perceptions of doing COPDMS with other student populations at different HE providers. Future researchers could also explore the long‐term effects of COPDMS. To extend research on COPDMS for transition, researchers could investigate the application of COPDMS for other student transitions such as the transition to postgraduate employment. To go beyond transition, future researchers could explore the effects of COPDMS on contemporary HE issues such as retention and dropout.

## CONCLUSION

Two separate samples of first‐year undergraduate students reported increased relational identification and perceived availability of social support after participating in COPDMS. Organizational identification and self‐efficacy for learning and performance remained unchanged in both studies. In Study 2, qualitative analyses resulted in five higher order themes relating to students’ perceptions of participating in a COPDMS intervention: (1) emotionality; (2) personal development; (3) storytelling; (4) enhanced group outcomes; and (5) task appropriateness and value. Overall, data provide evidence that COPDMS benefits individual and group‐level outcomes pertinent to students transitioning to a HE environment. Qualitative data also provides insights and considerations for those seeking to use PDMS in education.

## CONFLICT OF INTEREST

The authors have no conflict of interest to declare.

## AUTHOR CONTRIBUTION

**Andrew L. Evans:** Conceptualization; Data curation; Formal analysis; Investigation; Methodology; Writing – original draft; Writing – review & editing. **Matthew J. Slater:** Conceptualization; Writing – review & editing. **Martin J. Turner:** Conceptualization; Writing – review & editing.

## Data Availability

The data that support the findings of this study are available from the corresponding author upon reasonable request.
